# Constant illumination reduces circulating melatonin and impairs immune function in the cricket *Teleogryllus commodus*

**DOI:** 10.7717/peerj.1075

**Published:** 2015-07-16

**Authors:** Joanna Durrant, Ellie B. Michaelides, Thusitha Rupasinghe, Dedreia Tull, Mark P. Green, Therésa M. Jones

**Affiliations:** 1 School of BioSciences, The University of Melbourne, Melbourne, Victoria, Australia; 2 Metabolomics Australia, Bio21 Institute, The University of Melbourne, Melbourne, Victoria, Australia

**Keywords:** Circadian rhythm, Melatonin, Immune function, Invertebrate, Lysozyme-like activity, Constant illumination, Ecological light pollution, Light at night

## Abstract

Exposure to constant light has a range of negative effects on behaviour and physiology, including reduced immune function in both vertebrates and invertebrates. It is proposed that the associated suppression of melatonin (a ubiquitous hormone and powerful antioxidant) in response to the presence of light at night could be an underlying mechanistic link driving the changes to immune function. Here, we investigated the relationship between constant illumination, melatonin and immune function, using a model invertebrate species, the Australian black field cricket, *Teleogryllus commodus*. Crickets were reared under either a 12 h light: 12 h dark regimen or a constant 24 h light regimen. Circulating melatonin concentration and immune function (haemocyte concentration, lytic activity and phenoloxidase (PO) activity) were assessed in individual adult crickets through the analysis of haemolymph. Constant illumination reduced melatonin and had a negative impact on haemocyte concentrations and lytic activity, but its effect on PO activity was less apparent. Our data provide the first evidence, to our knowledge, of a link between exposure to constant illumination and variation in haemocyte concentration in an invertebrate model, while also highlighting the potential complexity of the immune response following exposure to constant illumination. This study provides insight into the possible negative effect of artificial night-time lighting on the physiology of invertebrates, but whether lower and potentially more ecologically relevant levels of light at night produce comparable results, as has been reported in several vertebrate taxa, remains to be tested.

## Introduction

Life evolved under a reliable daily cycle of light and dark, such that many, if not all, behavioural patterns and physiological processes are highly influenced by a predictable day-night (or circadian) rhythm (Moore-Ede, Sulzman & Fuller, 1982; Refinetti & Menaker, 1992; Czeisler & Klerman, 1999). Increasing evidence suggests that the presence of constant light can disrupt many aspects of circadian rhythm and have negative effects on physiology. In vertebrates, including humans, such physiological effects of constant exposure to light include disruptions to juvenile growth (Bruning, Hölker & Wolter, 2011), accelerated aging (Vinogradova et al., 2009; Bartsch, 2010), increased depression and anxiety (Ma et al., 2007; Fonken et al., 2009), an increased risk of cancer (Blask et al., 2003; Anisimov et al., 2004; Anisimov, 2006; Haim & Zubidat, 2015) and generally a negative impact on immune function (Maestroni, Conti & Pierpaoli, 1986; Moore & Siopes, 2000; Oishi et al., 2006). The absence of a circadian cue is likely to be a strong driver behind these effects; however, similar effects are evident in response to an extension in the number of hours of light exposure (Megdal et al., 2005; Blask, 2009; Stevens, 2009; Dickerman & Liu, 2012) or exposure to dim light during periods of natural darkness (Kloog et al., 2010; Spivey, 2010; Bedrosian et al., 2011; Aubrecht, Weil & Nelson, 2014; Borniger et al., 2014). Together these studies suggest that it may be the loss of darkness itself that promotes behavioural and physiological change (Longcore & Rich, 2004; Rich & Longcore, 2006; Gaston et al., 2013). Despite the recent surge in research on the impacts of exposure to constant illumination or light at night, the underlying mechanism promoting changes to physiology and especially immune function remains largely untested, particularly in invertebrate models.

An important mechanistic component of the circadian system that varies with exposure to light, and thus a proposed link between the presence of light and the observed negative effects on physiology, is the hormone melatonin (N-acetyl-5-methoxy-tryptamine) (Poeggeler, 1993; Reiter, 1993; Hardeland, Pandi-Perumal & Cardinali, 2006; Pandi-Perumal et al., 2006; Tan et al., 2010; Weil et al., 2015). Melatonin is typically produced during darkness and suppressed during daylight hours, resulting in a diurnal hormonal rhythm (Reiter, Tan & Fuentes-Broto, 2010). Melatonin appears to occur in all known taxa and its structure and biosynthesis are highly conserved between vertebrates and invertebrates (Poeggeler, 1993; Vivien-Roels & Pevet, 1993; Pandi-Perumal et al., 2006; Tan et al., 2010). While the principle function of melatonin is to relay information about changes in photoperiod (Zawilska, 1996; Arendt & Skene, 2005; Pandi-Perumal et al., 2006), it also plays an important role as a free radical scavenger and antioxidant (for recent reviews, see (Tan et al., 2010; Garcia et al., 2014)). At the cellular level, endogenous vertebrate melatonin is positively linked to cell maintenance (Garcia et al., 2014), mitochondrial activity (Reiter et al., 2003; Acuna-Castroviejo et al., 2007; Skulachev, 2009) and the increased antioxidant capacity of sperm and eggs (Benot et al., 1999; Chuffa et al., 2011). The indirect antioxidant effects and the links between melatonin and physiological processes such as immune function are less well understood mechanistically but nonetheless are proposed (Guerrero & Reiter, 2002; Pohanka, 2013).

Studies investigating the relationship between endogenous melatonin concentration and immune function in vertebrates demonstrate overwhelmingly that it has an immune-enhancing effect (Moore & Siopes, 2000; Brennan et al., 2002; Moore & Siopes, 2003; Srinivasan et al., 2005; Esteban et al., 2013), including recent evidence for a role in the innate immune response (Calvo, Gonzalez-Yanes & Maldonado, 2013; Shi et al., 2015; Weil et al., 2015). Given the ubiquitous presence of melatonin in the animal kingdom, as well as its conserved structure and functional versatility, it seems highly likely that it will be similarly important in invertebrates (Poeggeler, 1993; Vivien-Roels & Pevet, 1993; Pandi-Perumal et al., 2006; Jones et al., 2015). Further, while invertebrates lack an adaptive immune system, their innate immune system is highly effective and has cellular recognition and defensive pathways that are analogous with vertebrates (for a comprehensive coverage of the discipline see, Beckage, 2008). It is predicted that the potential for melatonin to act either directly or indirectly as a regulator of physiological processes or as an antioxidant will be compromised by constant light exposure, as nocturnal melatonin production is suppressed by light (Nurnberger et al., 1985; Pauley, 2004). Evidence suggests that even low levels of light (<1 lux) at night supress circulating melatonin concentrations (Arendt & Ravault, 1988; Dominoni et al., 2013). Experimental studies that have investigated the relationships between constant illumination, circulating melatonin concentration and immune function are limited and, to our knowledge, data from invertebrates are largely absent (but see, Jones et al., 2015).

In this study, we used the Australian black field cricket, *Teleogryllus commodus* as a model species to explore the relationships between constant illumination, circulating melatonin concentration and immune function. We had four main aims: (1) to assess whether the presence of constant illumination is correlated with variation in the concentration of endogenous circulating melatonin; (2) to determine whether the presence of constant illumination affected three independent measures of immune function; (3) to assess whether the patterns observed were comparable for males and females; and, (4) to assess whether melatonin concentration correlated with any of the immune parameters measured.

## Methods

Experimental crickets were third-generation, laboratory-adapted offspring of founders captured in Victoria, Australia (37.56238 S, 145.31920 E). Stock population crickets (approximately 250 per generation) were maintained in a climate-controlled laboratory (26 °C) under a 12 h light (4,000 Lux): 12 h dark (0 Lux) cycle. They were housed in plastic containers (320 mm length × 275 mm width × 200 mm depth) with egg cartons for shelter and *ad libitum* water and dried cat food (Friskies Senior; Rhodes, NSW, Australia), at a density of around 50 individuals per container.

*Rearing conditions*—To investigate the effect of constant exposure to light on circulating melatonin levels and immune function, crickets (from 37 breeding pairs) were reared from eggs through to five weeks of age post-adult emergence in one of two light regimens—12 h light: 12 h dark (as above; LD crickets) or 24 h constant light (LL crickets). Each breeding pair contributed eggs equally to each light regimen. Incubators set at a constant 28 °C (the optimal temperature for egg hatch in the laboratory) were used to provide the different regimens and were lit by white light florescent tubes (4,000 lux); roughly equivalent to indirect daylight. Individual incubator effects were eliminated by alternating the light regimen in each incubator weekly, and swapping the treatment crickets accordingly, ensuring each incubator contributed equally to each regimen, but crickets were always maintained on their designated light regimen. Within the rearing containers, light levels were 4,000 Lux; however upturned egg cartons provided a refuge (the underside was 400 Lux) within each container. It should also be noted that under our experimental LD regimen, crickets commenced singing one hour before incubator lights off (corresponding to the period they would normally commence singing in the wild). Thus, while LL crickets had no explicit photoperiod, they were exposed to an acoustic circadian cycle cue, as they were able to hear LD crickets when they commenced singing and indeed LL males responded by commencing singing accordingly. The lighting regimen (day-night versus constant illumination) utilised also mirrors that of previous laboratory studies (Moore & Siopes, 2000; Bruning, Hölker & Wolter, 2011).

From the third instar, juvenile crickets were housed at low densities (*n* = 3 crickets) in small containers (170 mm length × 120 mm width × 55 mm height) and were provided with *ad libitum* dried cat food, water and a row of egg cartons for shelter. Following the final juvenile moult, adult crickets were marked individually, separated by sex, and maintained at densities of three per container. At 14 ± 2 days, half the adult crickets were mated to individuals of the opposite sex within the same treatment group. Following this mating period, all females were separated into individual containers and given a sand pad to oviposit, as both mated and unmated females lay eggs (the latter unfertilised). Males were returned to their original container, in groups of three. Whether crickets were mated (males and females) was included in all statistical analyses (see statistics section below); mating data are reported in another study (EB Michaelides, J Durrant and TM Jones, 2015, unpublished data). Crickets were subsequently checked thrice weekly and maintained in their containers until the final haemolymph sample or death.

*Morphology*—Body weight (to the nearest mg) was taken following the final moult and prior to carrying out any procedure. Following death, both femurs were removed from an individual cricket; taped to a glass slide and then digitally photographed with a SPOT Flex camera system (Sterling Heights, MI, USA) mounted at × 50 magnification on an Olympus SZX7 stereomicroscope (Olympus; Tokyo, Japan). Femur dimensions were determined using Image J (Version 1.45S; NIH, Maryland, USA). The average length of the two femurs was used as a measure of body size (Carriere, Simons & Roff, 1996). As mass typically varies with body size in invertebrates, we used body condition (calculated from the residuals of a regression analysis between femur length and body weight at each procedural time point) rather than mass, as a more accurate measure of relative condition.

### Haemolymph collection and processing

Unless otherwise stated, all chemicals and reagents were purchased from Sigma-Aldrich (North Ryde, NSW, Australia). To control for potential variation in diurnal changes of enzymes and hormone concentrations, and to standardize sampling across treatments, all haemolymph samples were taken under the same immediate lighting conditions, during the “light” period, one to two hours prior to incubator “lights off” for LD crickets. It should be noted that while this is predicted to be the point when concentrations of melatonin are lowest (Reiter, 1993), we expect differences between the two treatment groups due to the chronic levels of exposure to light experienced by LL crickets. We further expect that any observed differences would be magnified if samples were taken during the natural “night” (Kezuka et al., 1988; Iigo et al., 1995; Porter et al., 1999). Following adult emergence, samples were collected thrice from the same individual during the study to provide a repeated assessment and to minimise the amount of haemolymph extracted on any given day. Sampling was undertaken at (i) 22 ± 1 day (3-week sample: used to assess baseline immune function), (ii) 24 ± 1 day (sample used to assess melatonin concentration) and (iii) 36 ± 1 day (5-week sample: used for second assessment of immune function). For each sample, a small puncture was made in the left side of the cricket abdomen using a 27G sterile needle (Becton Dickinson and Co.; Melbourne, VIC, Australia). This resulted in a small haemolymph bubble on the cuticle surface. For the 3- and 5-week samples, 2 µl of this haemolymph was collected using a micropipette and transferred to a 0.5 ml Eppendorf tube (Sarstedt, Mawson Lakes, SA, Australia) containing 16 µl of pre-prepared anticoagulant solution (100 mM NaOH, 150 mM NaCl, 22 mM EDTA, 45 mM citric acid in distilled water) maintained on ice. A further 6 µl of haemolymph was collected immediately afterwards, transferred to a 0.5 ml Eppendorf tube with 80 µl of Phosphate buffer saline (PBS; 11.9 mM phosphate, 137 mM NaCl, 2.7 mM KCl, pH 7.4), snap frozen in liquid nitrogen and then stored at −80 °C until later analysis for lytic activity and phenoloxidase level (see below). For the melatonin samples, a total of 10 µl of haemolymph was extracted as above, placed in a 0.5 ml Eppendorf tube with 10 µl of heparin to prevent coagulation, snap frozen in liquid nitrogen and then stored at −80 °C until analysis (see below). Crickets have a very efficient coagulation system, as small puncture wounds are common during fights or interactions, so crickets recovered immediately from these procedures and there were no obvious effects on survival.

### Variation in immune measures

The insect innate immune response is composed of both cellular and humoral defence pathways that interact to mount an effective immune response. We used three common immune measures to explore variation in immunocompetence in adult crickets: (i) The concentration of circulating haemocytes was calculated, as these are the cells that mediate the core cellular defence pathway in invertebrate innate immunity, including phagocytosis and encapsulation (Ribeiro & Brehélin, 2006); (ii) Lysozyme-like (herein referred to as lytic) activity was assessed as a measure of the capability of the humoral defence pathway. As lysozymes are a group of antibacterial enzymes principally involved in degrading bacterial cell walls, this assay specifically indicates the potential for crickets to resist bacterial challenges (Beckage, 2008); (iii) Phenoloxidase (PO) activity was assessed as a second measure of the humoral defence pathway, as PO becomes activated upon cuticular wounding or infection and is an important precursor instigating repair and encapsulation following infection or parasitism of larger foreign bodies (Kanost & Gorman, 2008). Each of these measures was assessed at two different time-points: three weeks after the final moult (3-week sample) and again two weeks later (5-week sample), following this initial wounding challenge (Siva-Jothy, Moret & Rolff, 2005; Moreno-Garcia et al., 2013). This provided us with a baseline immune assessment (3-week sample) and a post-immune challenge assessment (5-week sample).

(i) *Haemocyte concentration:* To determine the concentration of haemocytes, a 10 µl sample of the haemolymph-anticoagulant solution (see above) was placed onto a Neubauer haemocytometer (Blaubrand, Wertheim, Germany) at × 40 magnification on an Olympus BX50 stereomicroscope (Olympus, Tokyo, Japan). The number of haemocytes was counted and expressed as a concentration (Drayton & Jennions, 2011).

(ii) *Lytic activity:* Lytic activity and phenoloxidase assays were adapted from previously published methods (Bailey, Gray & Zuk, 2011; Drayton & Jennions, 2011). To assess lytic activity, 10 µl of haemolymph-PBS solution (described above) was added to 80 µl of *Micrococcus lutus (lysodeikticus)* bacteria solution (3 mg/ml PBS) in a round bottom 96-well plate (Sarstedt, Mawson Lakes, SA, Australia). Lysozymes in the haemolymph gradually degrade the bacteria in the solution causing the turbidity of the solution to decrease. Consequently, turbidity, as an indirect measure of lytic activity, was quantified by the absorbance measured at 490 nm using a microplate spectrometer (PerkinElmer Enspire 3.0 Multimode Plate Reader; Melbourne, VIC, Australia). Each plate was maintained at 30 °C and the change in absorbance measured over a 120 min period. Preliminary trials indicated that suspended particles settled over time, so the solution was thoroughly mixed prior to the final reading. The total change in absorbance was calculated as the initial absorbance reading minus the final reading, and these relative changes in absorbance were used to determine differences between treatments. Higher values indicate greater lytic activity. Each sample was run in duplicate and on each plate a series of controls were included; two blanks (air only), two sample controls (pure PBS in place of the haemolymph-PBS solution) and 12 PBQCs (pooled biological quality controls). The PBQCs consisted of two different pools of haemolymph that were pre-prepared and separated into appropriate aliquots in order to be able to include two of each PBQC at the start, the middle and the end of each plate. The mean intra- and inter-coefficients of variation for all lysozyme assays were 6.4% and 7.3% respectively (*n* = 13 assays).

(iii) *Phenoloxidase (PO) assay:* When in circulation in the haemolymph, PO is stored as an inactive precursor, pro-phenoloxidase. To measure the total PO present in cricket haemolymph, it was necessary to first cleave all pro-phenoloxidase with the proteolytic enzyme, *α*-chymotrypsin (Moreno-Garcia et al., 2013). For each sample, 5 µl of the haemolymph-PBS solution was added to 7 µl of 1.3 mg/ml bovine pancreas *α*-chymotrypsin in a well of a round-bottom 96-well plate and then incubated for 20 min at room temperature. Following incubation, 90 µl of L-dihyroxyphenylalanine (L-DOPA) was added. As PO converts L-DOPA to dopachrome it causes the solution to darken, thus, the more PO in the sample, the darker the solution becomes over time. Immediately after the addition of L-DOPA, the plate was loaded into the microplate spectrometer (as above) maintained at 26 °C and the change in absorbance at 490 nm measured over 120 min. The total change in absorbance was calculated as the final minus the initial absorbance reading, and these relative changes in absorbance were used to determine differences between treatments. Higher values indicate greater total phenoloxidase levels. Plate layout and the inclusion of controls was the same as for the lysozyme assay. The mean intra- and inter-coefficients of variation for all PO assays were 6.0% and 6.4% respectively (*n* = 14 assays).

### Measurement of circulating melatonin concentration

Total melatonin (free and protein-bound) was extracted with dichloromethane (DCM), as previously described (De Almeida et al., 2011). To each 20 µl haemolymph-heparin sample (described above) 100 µl of DCM was added, the sample vortexed for 5 min and centrifuged at 9,800 g, 4 °C, for 10 min. The lower DCM phase, with the extracted melatonin, was transferred to a new 1.5 mL Eppendorf tube. The top phase was re-extracted with a second 100 µl aliquot of DCM as above. The second DCM lower phase extract was pooled with the first extract and dried under a nitrogen atmosphere. The dried sample was resuspended in 50 µl of acetonitrile (ACN), vortexed for 15 min, centrifuged at 9,800 g, 4 °C for 10 min, and the supernatant transferred to a High Performance Liquid Chromatography (HPLC) sample vial (Agilent Technologies, Mulgrave, VIC, Australia). Pooled biological quality control samples (PBQC) were prepared by pooling the final supernatant of ten haemolymph samples to form a master mix and then re-aliquoted (25 µl) into HPLC sample vials to generate individual PBQC samples.

Melatonin concentrations in cricket haemolymph were measured using High Performance Liquid Chromatography-Mass Spectrometry (HPLC-MS), based on previously published methods (De Almeida et al., 2011; Ozkan et al., 2012), but highly adapted and optimised for the current experiment. Method development included recovery experiments that showed melatonin recovery in a human plasma matrix at 79% (at a spiked level of 100 pg/µl). A stock of melatonin standard curve was prepared using a 10 µg/ml melatonin stock in ACN, and serially diluted with ACN to generate nine different concentrations over the range 2.5 to 1,000 pg/ml. A composite standard curve, used to compare samples against, was derived from all standard curves run at the start, middle and end. After every tenth biological sample, PBQCs (*n* = 14) were run, as well as a melatonin QC standard at 100 pg/ml in ACN (*n* = 14). Blanks (ACN only) were also run after every fifth biological sample.

Melatonin in samples and standards were resolved by injecting 15 µl aliquots onto a 50 mm × 2.1 mm × 2.7 µm C18 column (Agilent Technologies, Mulgrave, VIC, Australia) using an Agilent LC1200 system with gradient of 0.1% of ammonium acetate in water and acetonitrile at a flow rate of 0.4 ml/min. Separated melatonin was detected by electrospray ionisation-mass spectrometry (ESI-MS) using an Agilent Triple Quad 6460 (Agilent Technologies) instrument. Multiple reaction monitoring (MRM) with the transitions m/z 233/174 and m/z 233/159, as quantifier and qualifier MRMs respectively, was used to detect the melatonin, in positive mass spectrometer mode (De Almeida et al., 2011). The MS parameters, capillary voltage, fragmentor voltage, and collision energy were 3,500 V, 130 V, and 5 V and 20 V, respectively. In all cases, the collision gas and sheath gas was nitrogen at a flow rate of 10 L/min and temperatures were maintained at 320 °C and 350 °C respectively. LCMS data was processed using Agilent MassHunter quantitative software, version 5 (Agilent Technologies). Assay sensitivity was determined as 25 pg/ml melatonin in cricket haemolymph solution.

### Statistical analysis

All analyses were performed in JMP 11.0 (SAS Institute, NC, USA). All data were assessed for normality using a Shapiro–Wilk test and transformed appropriately in order to meet the normality requirement; melatonin concentration was natural log transformed and haemocyte concentration, lytic activity and PO activity were square root transformed. Each immune parameter was analysed in two separate ways. First, differences at the 3-week sampling period were investigated to assess the effect of light regimen on baseline immune function. Second, the changes in each parameter from the 3-week to the 5-week period were compared to assess the effect of light regimen on the ability of the crickets to respond to the wounding challenge of the 3-week extraction. Data for melatonin concentrations were only included in the analysis if above the 25 pg/ml detection limit. To determine the effect of light regimen on immune function and melatonin concentration, we used generalised linear models (GLMs). Maximal models included light regimen, sex and mating status as categorical variables, and interactions were included where biologically appropriate. Each model was reduced using hierarchical removal of all terms with a significance of *P* > 0.1 (except our designated light regimen). Femur length and body condition were also included in all models as continuous variables, to incorporate possible variation in these traits. Unless otherwise stated, significant interactions were assessed using post-hoc Tukey’s HSD tests, and data presented are untransformed means ± SE. All tests were two-tailed with a significance level of *P* < 0.05. To assess whether melatonin concentration and our three immune measures were correlated we used the non-parametric Spearman’s rank correlation test on untransformed data. To reduce the likelihood of a type I error, we applied a Holm-Bonferroni correction for multiple tests and report only the corrected *P* values (Aickin & Gensler, 1996).

## Results

### Adult morphology

Adult femur length varied between light regimens (LD crickets =10.42 ± 0.10 mm, *n* = 165; LL crickets = 11.30 ± 0.06 mm, *n* = 135; *P* < 0.0001) and sexes (male crickets = 10.50 ± 0.12 mm, *n* = 119; female crickets = 11.02 ± 0.08 mm, *n* = 180; *P* < 0.0001). Body condition at the 3-week sampling period also differed between light regimens (LD crickets = 0.0002 ± 0.010; LL crickets = − 0.046 ± 0.009; *P* < 0.001) and sexes (male crickets = − 0.064 ± 0.008; female crickets = 0.008 ± 0.009; *P* < 0.001). The change in body condition from 3 to 5-weeks was comparable between both light regimens and sexes (LD crickets = 0.021 ± 0.008; LL crickets = 0.047 ± 0.013; P = 0.27). Size and body condition measures were initially included in all subsequent models but were removed where *P* > 0.10.

### Assessment of melatonin concentration

**Figure 1:**
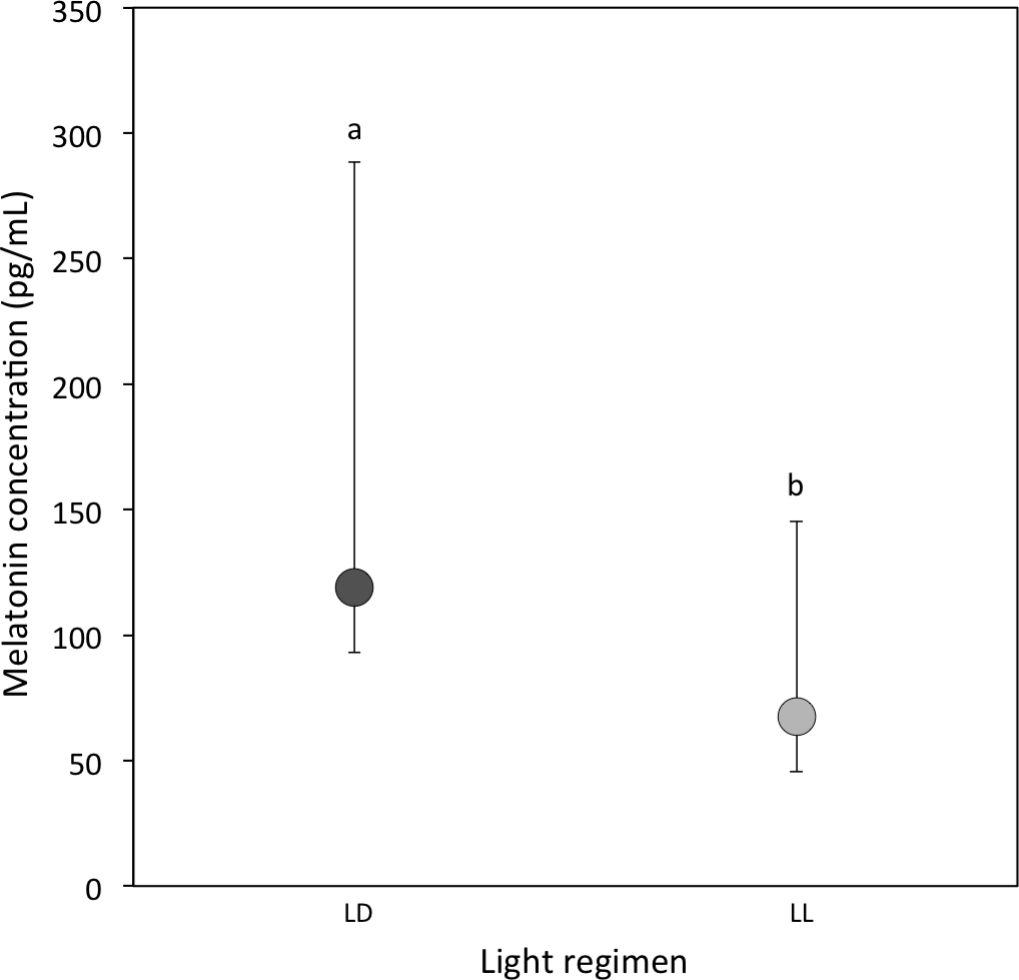
Circulating melatonin concentrations. Median circulating melatonin concentrations (pg/ml) of 22 day-old crickets, measured from haemolymph samples taken during the light period for LD (dark grey) and LL (light grey) crickets (LD *N* = 20; LL *N* = 10). Error bars indicate interquartile ranges about the median; different letters indicate significant differences between groups (*P* < 0.05).

A total of 20 out of 57 LD samples (35.1%; females = 9; males = 11) and 10 out of 44 LL samples (22.7%; females = 6; males = 4) were above the detectable assay limit of 25 pg/ml in haemolymph. A comparison of the experimental samples above 25 pg/ml showed that melatonin concentrations were higher in LD crickets compared to LL crickets (Ln transformed data: *P* = 0.03; Table 1D and Fig. 1).

**Table 1:** Models of 3-week immune function and melatonin. Generalized linear models exploring the effect of light regimen at the 3-week sampling period on: (A) haemocyte concentration, (B) lytic activity, (C) PO activityand (D) circulating melatonin concentration in adult crickets maintained in either a 12 h light: 12 h dark (LD) or constant light (LL) regimen. Values highlighted in bold are main effects and interactions that are significant (*P* < 0.05). Haemocyte concentrations, lytic and PO activity were square-root transformed; melatonin concentrations were natural log transformed prior to analysis.

Model parameters	*β* ± SE	Statistic	*P* value
**(A) 3-week haemocyte concentration (cells/ml × 10^6^)**			
*Whole model*		*F*_1,127_ = *0.11*	*0.74*
Light regimen		*F*_1,127_ = 0.11	0.74
**(B) 3-week lytic activity (Δ absorbance)**			
*Whole model*		*F*_4,113_ = *7.25*	<*0.001*
Light regimen		*F*_1,116_ = 7.40	**0.008**
Sex		*F*_1,116_ = 7.23	**0.008**
Light regimen × Sex		*F*_1,116_ = 6.57	**0.01**
3-week body condition	0.40 ± 0.17	*F*_1,116_ = 5.65	**0.02**
**(C) 3-week PO activity (Δ absorbance)**			
*Whole model*		*F*_2,127_ = *14.99*	<*0.001*
Light regimen		*F*_1,128_ = 2.15	0.15
Sex		*F*_1,128_ = 14.54	<*0.001*
**(D) Melatonin concentration (pg/mL)**			
*Whole model*		*F*_3,26_ = *2.43*	*0.09*
Light regimen		*F*_1,26_ = 5.07	**0.03**
Sex		*F*_1,26_ = 1.32	0.26
Light regimen∗Sex		*F*_1,26_ = 2.83	0.10

### Variation in immune function

**Figure 2:**
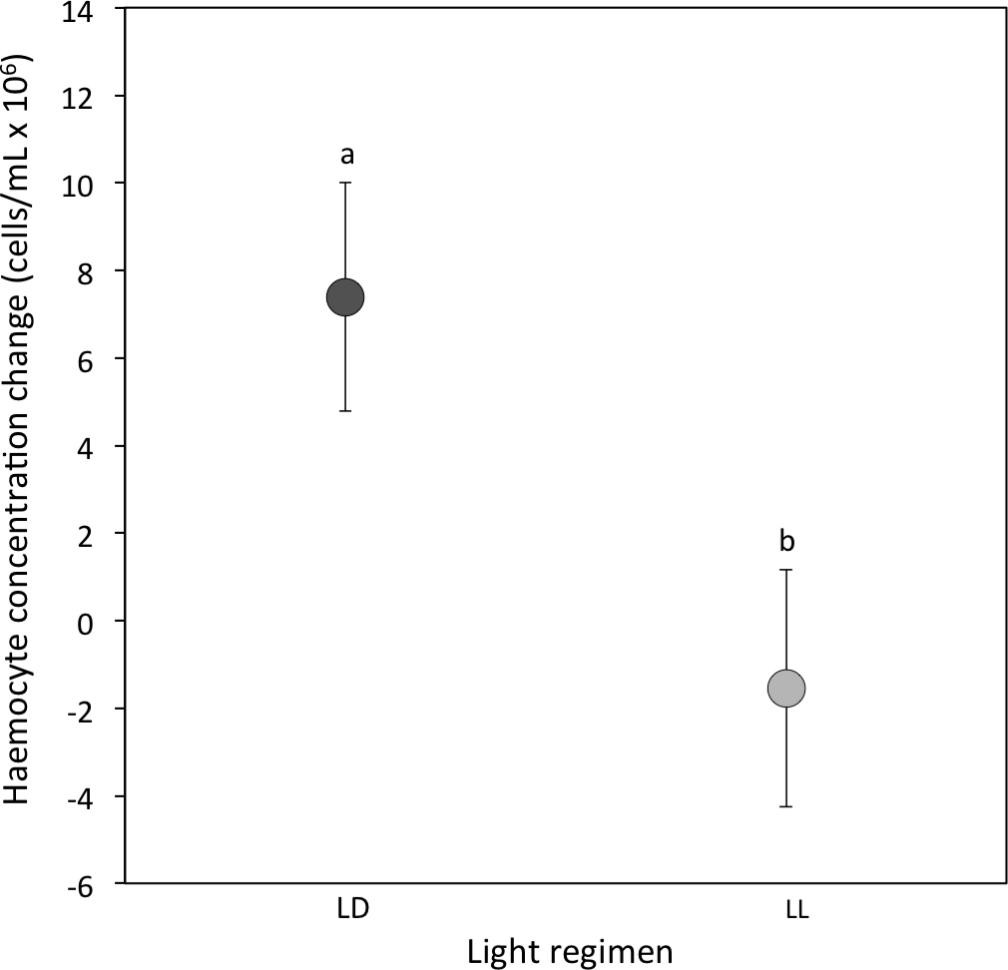
Haemocyte concentration change. Mean change in haemocyte concentration (cells/ml × 106) from the 3-week to the 5-week sampling period for LD (dark grey) and LL (light grey) crickets (LD *N* = 53; LL *N* = 37). Error bars indicate standard errors (SE) about the mean; different letters indicate significant differences between groups (*P* < 0.05).

(i) *Haemocyte concentration:* At the 3-week sampling period haemocyte concentrations were comparable between light regimens (median [interquartile range] for LD crickets = 15.68 [8.68–22.36]; LL crickets = 16.04 [8.58–22.52]; Sqrt transformed data: *P* = 0.74; Table 1A). However, the change in haemocyte concentration from the 3 to 5-week sampling period was significantly different between LD and LL crickets. Haemocyte concentrations in LD crickets increased between the 3 and 5-week sampling periods, but decreased in LL crickets (*P* = 0.007; Table 2A and Fig. 2). The change in haemocyte concentration between weeks 3 and 5 was also positively correlated with the change in body condition (*P* = 0.003; Table 2A).

**Table 2:** Models of the immune function change. Generalized linear models exploring the changes from the 3-week to 5-week sampling period in the immune parameters: (A) haemocyte concentration, (B) lytic activity and (C) PO activity in adult crickets maintained in either a 12 h light: 12 h dark (LD) or constant light (LL) regimen. Values highlighted in bold are main effects and interactions that are significant (*P* < 0.05). Haemocyte concentrations, lytic activity and PO activity were square-root transformed prior to analysis.

Model parameters	*β* ± SE	Statistic	*P* value
**(A) Change in haemocyte concentration (cells/ml × 10^6^)**			
*Whole model*		*F*_2,84_ = *2.08*	*0.001*
Light regimen		*F*_1,85_ = 7.77	**0.007**
Change in body condition	77.90 ± 23.85	*F*_1,85_ = 9.09	**0.003**
**(B) Change in lytic activity (Δ absorbance)**			
*Whole model*		*F*_1,79_ = *0.09*	*0.77*
Light regimen		*F*_1,79_ = 0.09	*0.77*
**(C) Change in PO activity (Δ absorbance)**			
*Whole model*		*F*_3,89_ = *0.80*	*0.13*
Light regimen		*F*_1,91_ = 0.24	0.63
Mating status		*F*_1,91_ = 6.56	**0.002**

(ii) *Lytic activity:* At the 3-week sampling period, there was a significant interaction between light regimen and sex (*P* = 0.01; Table 1B; Fig. 3). LD crickets exhibited higher lytic activity than LL crickets (Sqrt transformed data: *P* = 0.008; Table 1B; Fig. 3) and females had higher lytic activity compared to males (Sqrt transformed data: *P* = 0.008; Table 1B and Fig. 3), while post-hoc Tukey’s HSD tests revealed that these differences were due to the higher lytic activity of LD females compared to all other groups (see Fig. 3). Lytic activity was also positively correlated with 3-week body condition (*P* = 0.02: Table 1B). Both LD and LL crickets showed a similar increase in lytic activity from the 3 to 5-week sampling period (LD crickets = 0.074 ± 0.026; LL crickets = 0.081 ± 0.036; *P* = 0.77: Table 2B).

**Figure 3:**
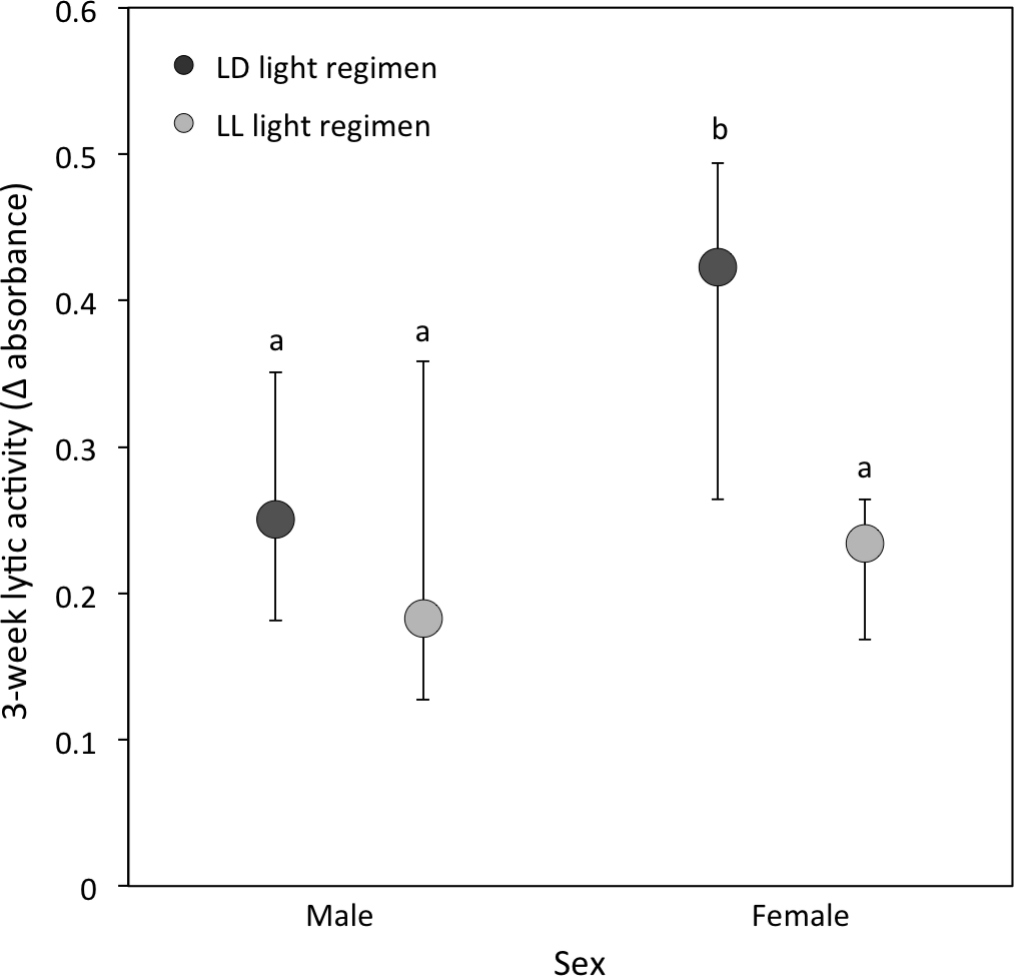
Lytic activity at the 3-week sampling period. Median lytic activity (Δ absorbance) of each sex for LD (dark grey) and LL (light grey) crickets (LD female *N* = 40, LD male *N* = 30, LL female *N* = 36, LL male *N* = 17). Error bars indicate interquartile ranges about the median; different letters indicate significant differences between groups (*P* < 0.05).

(iii) *PO activity*: This was comparable between light regimens at the 3-week sampling period (median [interquartile range] for LD crickets = 1.02 [0.70–1.40]; LL crickets = 1.09 [0.84–1.26]; Sqrt transformed data: *P* = 0.15; Table 1C), but differed significantly between the sexes (median [interquartile range] for males = 0.79 [0.55–1.15]; females = 1.11 [0.95–1.58]; Sqrt transformed data: *P* < 0.001; Table 1C). LD and LL crickets showed a comparable decrease in PO activity from the 3 to 5-week sampling period (LD crickets = −0.215 ± 0.059; LL crickets = −0.358 ± 0.079; *P* = 0.63). The change in PO activity varied with mating status: mated individuals had a larger decline in PO activity than unmated individuals (*P* = 0.002; Table 2C).

### Correlations among immune indicators

Haemocyte concentration was positively related to lytic activity (Spearman’s correlation: *r_s_* = 0.54, *P* < 0.0003, *n* = 202), and weakly positively related to PO activity (*r_s_* = 0.16, *P* = 0.04, *n* = 216). Lytic and PO activity however, were unrelated (*r_s_* = − 0.05, *P* = 0.49, *n* = 217).

### Correlation between melatonin and immune indicators

*LD crickets*—Melatonin concentration did not directly correlate with haemocyte concentration, lytic activity or PO activity at either the 3-week or 5-week sampling periods (*P* > 0.10 for all Spearman rank correlations).

*LL crickets*—Melatonin concentration was positively related to PO activity at the 3-week (Spearman’s correlation: *r_s_* = 0.73, *P* = 0.02, *n* = 11) but not the 5-week sampling period. All other correlations between melatonin concentration and immune parameters were non-significant (*P* > 0.10).

## Discussion

This study had four key findings. First, even when melatonin was predicted to be at its lowest diurnal concentration, crickets reared under constant illumination had a reduced circulating melatonin concentration compared to crickets reared under a diurnal (12 h light: 12 h dark) light cycle. Second, exposure to constant illumination had a significant impact on two of the three immune function indicators (haemocyte concentration and lytic activity, but not PO activity). Third, we identified sex differences in lytic and PO activity, while haemocyte and melatonin concentrations were comparable for males and females. Finally, we determined limited evidence for a link between immune function and melatonin concentration, although this may be a result of low sample size or sampling period.

### The effect of constant illumination on circulating melatonin concentrations

Our findings support the key prediction that constant illumination is correlated with a reduction in circulating melatonin concentrations, adding to the growing body of literature exploring the negative effect of light exposure on melatonin synthesis (Lewy et al., 1980; Brainard et al., 1982; Nurnberger et al., 1985; Moore & Siopes, 2000; Vera et al., 2005; Firth, Belan & Kennaway, 2006; Figueiro et al., 2011; Moore & Menaker, 2011). It should be noted that, to allow a direct comparison of baseline measures, we measured melatonin concentrations during the light period for both regimens. We therefore expect absolute concentrations (and the relative difference in concentration between each light regimen) to be at their lowest point, as has been demonstrated for other invertebrates (Vivien-Roels & Pevet, 1993; Hardeland & Poeggeler, 2003). Consequently, it is postulated the cumulative differences in melatonin concentrations over a 24 h period are likely substantially higher (Reiter, 1993; Iigo et al., 1995; Porter et al., 1999; Pandi-Perumal et al., 2006) and thus the biological implications may be substantially greater.

### The effect of constant illumination on immune function

Haemocyte concentration, lytic activity and phenoloxidase (PO) activity have been used as proxies for immune-competence in a range of invertebrate studies (Simmons & Roberts, 2005; Siva-Jothy, Moret & Rolff, 2005; Haine et al., 2008a; Haine et al., 2008b; Bailey, 2011; Bailey, Gray & Zuk, 2011; Drayton & Jennions, 2011; Drayton et al., 2012; McNamara et al., 2013). In contrast, the link between the presence of light and changes to immune function is rarely highlighted in any species other than vertebrates (Kirby & Froman, 1991; Moore & Siopes, 2000; Oishi et al., 2006; Bedrosian et al., 2011; Fonken, Haim & Nelson, 2012; Aubrecht, Weil & Nelson, 2014). Our measures of immune function varied in their response across the two light regimens but nonetheless broadly show a decline in response to the presence of constant illumination. Both haemocyte concentrations and lytic activity were negatively impacted in crickets maintained under constant illumination. The observed variation in haemocyte concentrations, which are thought to represent the core cellular response in invertebrate immunity (Ribeiro & Brehélin, 2006), is perhaps the most intriguing. Both LD and LL adult crickets had similar initial haemocyte concentrations despite the fact that they had been reared as juveniles under these differing light environments and thus one might predict that they would already exhibit variation in immune function. The subsequent up-regulation of haemocytes observed in LD, but not LL, crickets following the first wounding challenge (i.e., the 3-week sampling event) suggests that under relatively benign conditions LL crickets have the capacity to maintain normal cellular activity, however future immune challenges are likely to be more detrimental and thus might impact fitness disproportionately compared to crickets reared under a normal day-night light cycle. Similar decreases in analogous cellular components of the immune system in response to increased illumination at night have been reported for vertebrates (Moore & Siopes, 2000; Navara & Nelson, 2007; Bedrosian et al., 2011). The current study is however the first to show changes in haemocyte concentration in response to constant illumination in invertebrates.

The observed relationship between lytic activity and exposure to light at night appears more complex but is in alignment with previous findings (Jones et al., 2015). Both LL and LD crickets responded with increased lytic activity following the initial wounding challenge (Table 1B). We are unable to discount the possibility that this increase in lytic activity is age rather than wounding-related, although typically, immune-senescence is reported in other related cricket species (Adamo, Jensen & Younger, 2001; Park, Kim & Stanley, 2011). Nonetheless, the fact that individuals from both light environments have a similar response in lytic activity suggests that, while the core cellular response (haemocyte concentration) may be compromised in LL crickets following constant exposure to light, some degree of immunocompetence remains, at least in the relatively benign laboratory environment, with *ad libitum* food and water. How this translates into variation in fitness remains to be tested. Our data also indicate a sex difference with respect to the impact of light regimen on lytic activity: LD males had higher overall levels of lytic activity than all other groups. We can only speculate how and why this difference between the sexes arises. One possibility is that females invest more heavily in offspring production than males and trade-off immune function with other life history traits (Lochmiller & Deerenberg, 2000; Gwynn et al., 2005; Simmons & Roberts, 2005; Hegemann et al., 2013; McNamara et al., 2013; Reavey et al., 2014; Telemeco & Kelly, 2014). Although short-term female fecundity (to five weeks) was not measured in this study, lifetime female fecundity (number of eggs laid) in this experimental population tended to be higher for LD females compared to LL females (lifetime fecundity of LL females = 420.31 ± 64.64 eggs laid; *n* = 16; LD females = 597.39 ± 79.39 eggs laid; *n* = 18; *P* = 0.09; EB Michaelides, J Durrant and TM Jones, unpublished data). Thus, LD females may have been able to invest more heavily in egg laying yet still maintain a comparable capacity to respond to a bacterial infection as LL females.

In contrast to the significant impacts on haemocyte concentration and lytic activity, constant illumination had no detectable impact on PO activity. Several mutually non-exclusive explanations may exist for these observations. First, the PO pathway in *T. commodus*, which is involved in repair, encapsulation and melanisation (Kanost & Gorman, 2008), may be less susceptible to the presence of continuous light. It is also conceivable that crickets are able to trade-off maintenance in cuticular melanisation with investment in immune function as has been suggested for other biological stressors and immune challenges (Stanley, Miller & Tunaz, 2009; Bailey, 2011; Gonzalez-Santoyo & Cordoba-Aguilar, 2012). It is also possible that our assay, which measured the change in absorbance (and thus the change in PO activity) over a defined period, did not capture differences in absolute PO concentrations, merely changes in relative concentrations. Finally, we used a specific enzyme (*α*-chymotrypsin) to mobilise PO from pro-PO *in vitro* and assumed that this was representative of how individuals would actually respond *in vivo*. While there was no effect of the presence of light on PO activity, it did decline significantly between the 3-week and 5-week sampling periods and was higher for females compared to males. These lower PO levels in males are explained in other invertebrates as a result of physiological trade-offs including other aspects of immunity, differing investment in melanisation and courtship activity (Adamo, Jensen & Younger, 2001; Bailey, 2011; Gonzalez-Santoyo & Cordoba-Aguilar, 2012; Hangartner et al., 2013; Pinera et al., 2013). In *T. commodus*, males engage in mating and courtship songs which may necessitate an immune trade-off (Drayton et al., 2012). A final observation is the lack of concordance in response of all the immune parameters measured. This is perhaps not surprising given the known level of species- and sex-specific variation reported in invertebrates (for a review, see Siva-Jothy, Moret & Rolff, 2005) but it should be noted that they are in broad agreement with previous studies in *T. commodus* (Drayton et al., 2012).

### The role of melatonin in immune function and as an antioxidant

We found little evidence to support a direct link between variation in melatonin concentration and three potential correlates of immune function and are thus unable to draw any firm conclusions regarding the proposed role of melatonin as a mechanistic link between exposure to light at night and immune function (Maestroni & Conti, 1993; Pandi-Perumal et al., 2006; Tan et al., 2010). A possible reason, highlighted previously, for the lack of correlation in our study is that, to ensure consistency in samples across the two light regimens, melatonin measurements were taken from samples extracted in the light. This is the lowest point in the daily melatonin cycle and when variation between individuals in the two light regimens is predicted to be least. We also did not determine exactly how immune function was modulated and the lack of concordance in our three measures of immune capacity may in fact highlight the complexity of the underlying relationships between light, melatonin and life history traits, including immune function. Third, due to the logistic challenges of obtaining sufficient quantity for analysis, melatonin assays were conducted once only and thus, while we can correlate baseline melatonin levels, these were temporally out of sync with the immune assays (by only two days for the 3-week sample but nearly three weeks for 5-week sample—which is where we saw increasing difference in immune response between the two treatments). Finally, due to the low concentrations of circulating melatonin, we had a limited number of individuals with both melatonin and immune measures. Supplementation experiments with dietary melatonin have provided the strongest physiological support to date for the impact of constant light and the immune enhancing effects of melatonin in vertebrates (Moore & Siopes, 2000; Moore & Siopes, 2003; Vinogradova & Anisimov, 2013). Moreover, recent evidence from a supplementation experiment in *T. commodus* confirms that the provision of dietary melatonin may partially mitigate the detrimental immune effects of constant illumination (Jones et al., 2015). Further studies are required to confirm the generality of these results under dimmer and possibly more relevant lighting conditions.

### Melatonin and immune function in the context of ecological light pollution

The presence of increasing intensities of artificial light at night in urban areas is becoming a growing ecological problem (Cinzano, Falchi & Elvidge, 2001; Chepesiuk, 2009; Gaston, Visser & Hölker, 2015). Many species living in or near urban areas no longer experience any period of “true darkness” (Longcore & Rich, 2004), and the levels of urban night lighting are unlikely to ever return to those under which life evolved. Even many protected natural areas such as national parks are no longer free from the presence of light at night (Bennie et al., 2015; Gaston, Duffy & Bennie, 2015). Increasing evidence suggests that in vertebrates nighttime light levels as low as 5 Lux (or less)—even lower than residential street lighting - can result in significantly reduced night-time melatonin concentrations (Brainard et al., 1982; Zachmann et al., 1992) and lead to compromised immune function (Bedrosian et al., 2011). The impact of night lighting has the capacity to be significantly worse for nocturnal species, such as *T. commodus*, particularly if the presence of light compromises an individual’s ability to respond to minor wounding challenges, such as those inflicted in this study. If a reduction in immune capacity leads to significant declines in other life history traits, including survival and reproductive success, then this would have severe implications for individual and species fitness (Hölker et al., 2010). It should be noted that the physiological (and behavioural) consequences of compromised immune function are likely to be far greater in a malign natural environment compared to the relatively benign laboratory setting, due to the greater number of immune challenges and lower availability of quality food resources.

## Conclusion

In this study we have demonstrated that constant illumination is linked to a low circulating melatonin concentration and that it has a negative impact on immune parameters; haemocyte concentration and lytic activity in the invertebrate *T. commodus*. The fact that we found limited evidence for a direct correlation between melatonin concentrations and immune function highlights the likely complex nature of their potential interactions. Gaining an understanding of how all species, both vertebrates and invertebrates, respond to varying exposure to light at night, the mechanisms underpinning these responses, as well as disentangling the complex relationships between life-history and immune function trade-offs will be critical if we are to maintain the health of urban environments where the presence of constant light is inevitable.

## Supplemental Information

10.7717/peerj.1075/supp-1 Supplemental Information 1 Durrant et al.dataset
